# Relatively Recent Evolution of Pelage Coloration in Colobinae: Phylogeny and Phylogeography of Three Closely Related Langur Species

**DOI:** 10.1371/journal.pone.0061659

**Published:** 2013-04-17

**Authors:** Zhijin Liu, Boshi Wang, Tilo Nadler, Guangjian Liu, Tao Sun, Chengming Huang, Qihai Zhou, Jiang Zhou, Tengcheng Que, Ziming Wang, Christian Roos, Ming Li

**Affiliations:** 1 Key Laboratory of Animal Ecology and Conservation Biology, Institute of Zoology, Chinese Academy of Sciences, Beijing, China; 2 Primate Genetics Laboratory, German Primate Center, Leibniz Institute for Primate Research, Göttingen, Germany; 3 Graduate School of the Chinese Academy of Sciences, Beijing, China; 4 Frankfurt Zoological Society, Endangered Primate Rescue Center, Cuc Phuong National Park, Ninh Binh Province, Vietnam; 5 College of Life Sciences, Guangxi Normal University, Guilin, China; 6 Gene Bank of Primates, German Primate Center, Leibniz Institute for Primate Research, Göttingen, Germany; 7 Guangxi Rescue and Research Center of Rare and Endangered Wild Animals, Guangxi Zhuang Autonomous Region Forestry Department, Nanning, China; 8 College of Animal Science, South China Agricultural University, Guangzhou, China; University of Florida, United States of America

## Abstract

To understand the evolutionary processes leading to the diversity of Asian colobines, we report here on a phylogenetic, phylogeographical and population genetic analysis of three closely related langurs, *Trachypithecus francoisi*, *T. poliocephalus* and *T. leucocephalus*, which are all characterized by different pelage coloration predominantly on the head and shoulders. Therefore, we sequenced a 395 bp long fragment of the mitochondrial control region from 178 *T. francoisi*, 54 *T. leucocephalus* and 19 *T. poliocephalus* individuals, representing all extant populations of these three species. We found 29 haplotypes in *T. francoisi,* 12 haplotypes in *T. leucocephalus* and three haplotypes in *T. poliocephalus*. *T. leucocephalus* and *T. poliocephalus* form monophyletic clades, which are both nested within *T. francoisi*, and diverged from *T. francoisi* recently, 0.46-0.27 (*T. leucocephalus*) and 0.50-0.25 million years ago (*T. poliocephalus*). Thus, *T. francoisi* appears as a polyphyletic group, while *T. leucocephalus* and *T. poliocephalus* are most likely independent descendents of *T. francoisi* that are both physically separated from *T. francoisi* populations by rivers, open sea or larger habitat gaps. Since *T. francoisi* populations show no variability in pelage coloration, pelage coloration in *T. leucocephalus* and *T. poliocephalus* is most likely the result of new genetic mutations after the split from *T. francoisi* and not of the fixation of different characters derived from an ancestral polymorphism. This case study highlights that morphological changes for example in pelage coloration can occur in isolated populations in relatively short time periods and it provides a solid basis for studies in related species. Nevertheless, to fully understand the evolutionary history of these three langur species, nuclear loci should be investigated as well.

## Introduction

Primates exhibit striking examples of skin and pelage color variation. Closely related species often exhibit marked color differences, especially in Colobinae [1]. We know little about how this color variation is generated and maintained by the processes of evolution. Whether morphological changes as for example in pelage coloration evolve rapidly in isolated population or a long time to accumulation is necessary, has not yet been determined.

Colobine monkeys (subfamily Colobinae) are a diverse group of Old World primates with 59–78 species grouped in up to 10 genera [2]–[4]. Extant colobines are found in a wide range of forest and woodland habitats in Africa and Asia. The Asian colobines are undoubtedly more diverse numerically and morphologically, and comprise 55 species across seven genera (*Pygathrix*, *Rhinopithecus*, *Nasalis*, *Simias, Presbytis*, *Trachypithecus* and *Semnopithecus*) [4]. The genus *Trachypithecus* comprises up to 20 species, which are grouped into various species groups according to similarities in morphology, ecology, behavior, distribution and genetics [2]–[8]. One of these species groups is the “limestone” or *Trachypithecus francoisi* langur species group [2], [4], [6]. The three northernmost species of the group, *Trachypithecus francoisi, T. poliocephalus* and *T. leucocephalus* occur in nearby distribution areas, but differ extremely in pelage coloration, and are thus a good example to study evolutionary processes of pelage coloration variation in colobine monkeys.

**Figure 1:**
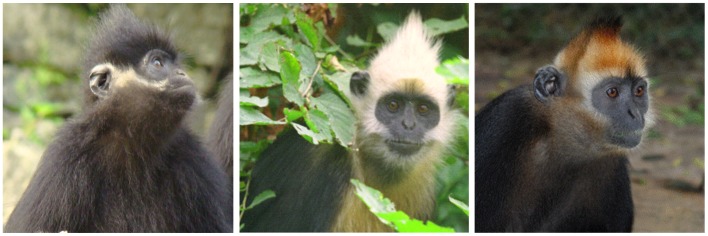
Head and shoulder coloration of François’s langur (*Trachypithecus francoisi*; left), white-headed langur (*T. leucocephalus*; middle) and golden-headed langur (*T. poliocephalus*; right).

**Figure 2:**
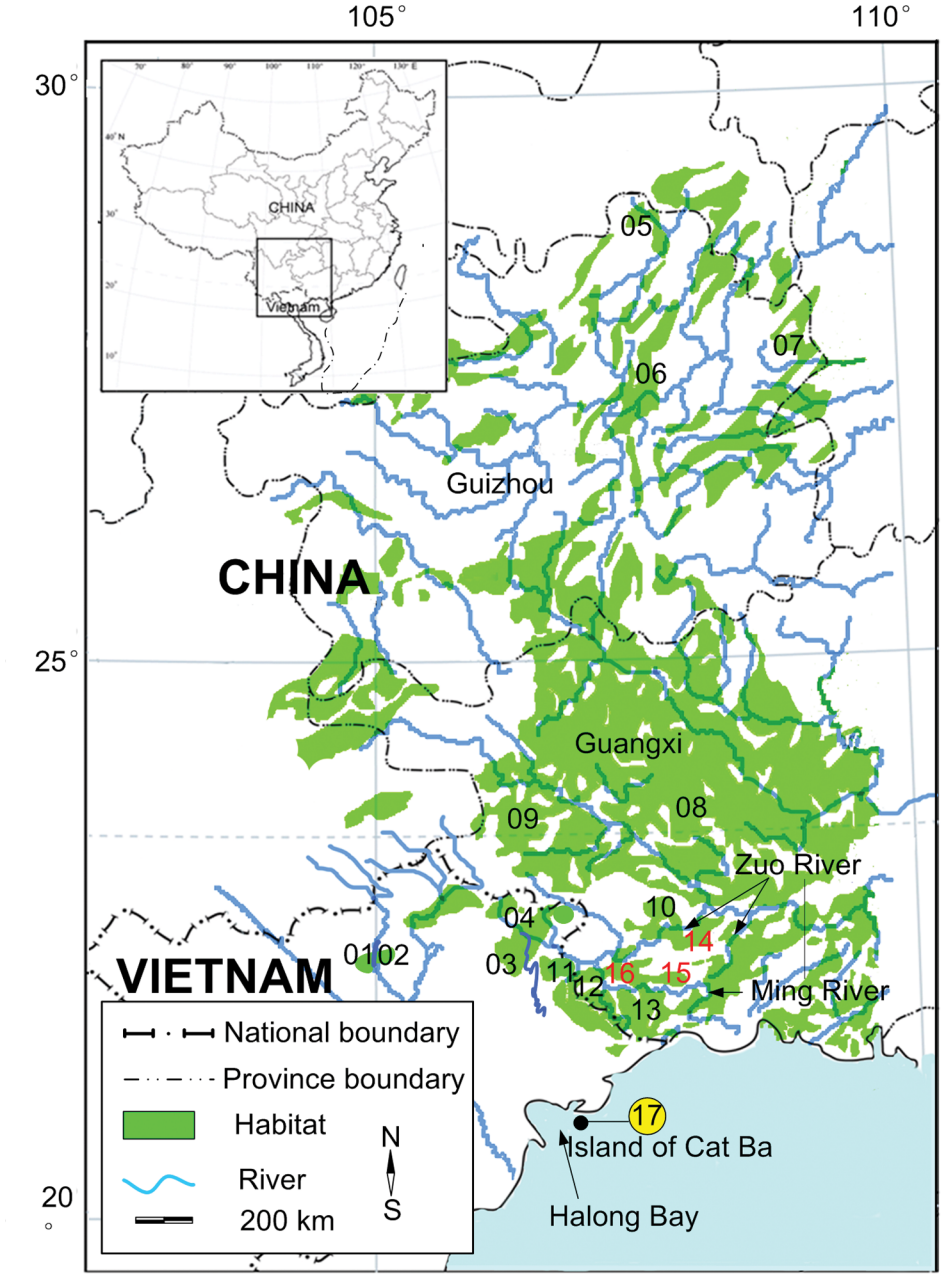
Distribution and sampling sites of *T. francoisi* (Lots 01–13), *T. leucocephalus* (Lots 14–16) and *T. poliocephalus* (Lot 17).

The François’s langur (*T. francoisi*, Figure 1) is endemic to karst hills in tropical and subtropical south-western China (Guizhou and Guangxi Province) and northern Vietnam (Figure 2). The species is medium-sized with mostly black silky hair and only white sideburns on cheeks. The golden-headed or Cat Ba langur (*T. poliocephalus*, Figure 1) occurs only on the island of Cat Ba, 30 kilometers off the coast of Hai Phong in Halong Bay, north-eastern Vietnam (Figure 2). As indicated by its common name, head and neck down to the shoulders are bright golden to yellowish. The white-headed langur (*T. leucocephalus*, Figure 1) is distributed within a narrow range in Guangxi Province, China, surrounded by *T. francoisi* populations (Figure 2). It has a similar coloration as *T. poliocephalus*, but with its head, crest hair, neck, upper shoulder and tail tip white.

As the habitats of *T. poliocephalus* and *T. leucocephalus* are close to or nested within the range of *T. francoisi* (Figure 2), it is hypothesized that *T. poliocephalus* and *T. leucocephalus* might have become isolated local populations of *T. francoisi* with different pelage colorations [9]–[11]. This would imply that *T. leucocephalus* and *T. poliocephalus* derived from *T. francoisi* independently and that the two former do not share a common ancestor (hypothesis 1). On the other side, it can be hypothesized that *T. leucocephalus* and *T. poliocephalus* share a common ancestor because both are more similar in pelage coloration than either species is to *T. francoisi* (hypothesis 2) [2], [7], [12]–[14].

To assess both hypotheses, we have analyzed sequence variation in the hypervariable region I (HVI) of the mitochondrial control region of *T. francoisi*, *T. leucocephalus* and *T. poliocephalus*, with the aim to (1) elucidate the phylogenetic, phylogeographical and demographical history of these three taxa, (2) detect which and when physical barriers isolated these relatively large-bodied primates, (3) evaluate the level and partitioning of genetic variation within and among them, and (4) describe phylogeographic relationships among extant populations and identify factors influencing genetic divergence and phenotypical differences. Although we used only a maternally inherited marker, we predict that our findings will provide deep insights into the evolutionary history of these three species, which are characterized by female philopatry [15], [16].

## Materials and Methods

### Ethics Statement

Our work was conducted according to relevant Chinese, Vietnamese and international guidelines, including countries where we analyzed samples. Approval for sample collection in the wild was obtained from the China Wildlife Conservation Association, Chinese State Forestry Administration and the Vietnamese National Forest Protection Department. Fecal samples from 165 wild animals were collected non-invasively without disturbing, threatening or harming the animals during field surveys. Twenty-seven tissue samples were obtained from deceased individuals found in the wild. Hair samples from 36 *T. francoisi* individuals were provided by Guangxi Wuzhou Inbreeding Center, and 20 and three frozen blood samples of *T. francoisi* and *T. leucocephalus* were provided by Nanning Zoo and Shanghai Safari Park, respectively. Blood collection and invasive plucking of hairs was performed during routine health checks. In Guangxi Wuzhou Inbreeding Center, Nanning Zoo and Shanghai Safari Park, family units were housed in single indoor cages (4–6 m×5–6 m×6 m). Outdoor play cages for 3–5 family units are equipped with swing and trees. Langurs were fed with fruits, vegetables and leaves, which they were used to eat in the wild three times per day; thus, they were never deprived of water or food. Periodically, they obtained multi-vitamine supplement. Samples were collected by institutional staff and directors gave permission to use them in our study. Collection of fecal and blood samples adhered to the American Society of Primatologists (ASP) Principles for the Ethical Treatment of Non-Human Primates (see www.asp.org/society/resolutions/EthicalTreatmentOfNonHumanPrimates.cfm).

### Sample Collection, DNA Extraction and Individual Identification

Twenty-three blood, 27 muscle, 36 hair and 165 fecal samples of different individuals (178 *T. francoisi*, 54 *T. leucocephalus*, 19 *T. poliocephalus*) from 17 forest lots (lots 1–17) representing all of the extant populations of these three langurs were collected (Figure 2, Table S1). Blood samples were stored at 0°C in acid citrate dextrose (ACD) solution B [17] until they were banked at −80°C. Hairs were stored under dry conditions in plastic bags. Muscle samples from deceased individuals found in the wild were stored in 95% ethanol. Fecal samples were collected during direct behavioral observations in the wild and stored in 95% ethanol. To avoid resampling of the same individual, each dropping was distinguished by freshness, size, shape and color, and feces found less than 1.5 m apart were not sampled [18]. DNA from blood and muscle samples was extracted using the standard PCI (25∶24:1 mix of phenol, chloroform, and isoamyl-alcohol and chloroform) method [19], while DNA from hair and feces was extracted with the Chelex-100 method [20] and the DNA Stool Mini Kit (Qiagen), respectively.

### Amplification and Sequencing of Mitochondrial DNA

A 395 bp long fragment of the HVI region was amplified and sequenced with the primers 5′-AAC TGG CAT TCT ATT TAA ACT AC-3′ and 5′-ATT GAT TTC ACG GAG GAT GGT-3′. Amplification was performed in a total volume of 50 µl containing 50 mM KCl, 10 mM Tris-HCl, 1.5 mM MgCl_2_, 200 µmol dNTPs, 0.2 µmol of each primer, 1 µg/µl BSA, 1.5 U Hotstart Taq DNA polymerase (Qiagen), and approx. 10 ng total DNA extract. Forty cycles were run on a Perkin-Elmer Cetus 9700 DNA thermocycler with pre-denaturing at 95°C for 15 min; denaturing at 95°C for 1 min, annealing at 56°C for 1 min, extension at 72°C for 1 min; and a final 10 min extension step at 72°C. Positive (DNA extracted from blood) and negative (water) controls were used to check PCR performance and contamination [21]. PCR products were purified with the QIAquick PCR purification Kit (Qiagen) and sequenced with the PrismTM BigDye Terminator Ready Reaction kit (Applied Biosystem Inc.) on an ABI 377 or 3130*xL* Genetic Analyzer. To avoid errors in amplification and sequencing, PCR amplifications of all samples were performed twice or more, and products were sequenced from both strands.

### Excluding “numts” and Cross-species Contamination

To exclude contaminations of the dataset with nuclear integrations of mitochondrial fragments (“numts”), we mainly used material in which nuclear DNA is highly degraded (feces) [22]. Moreover, from several specimens, two material types (hairs, feces) were available, which resulted in identical sequences and no multiple amplifications of different copies were detected by direct sequencing of PCR products. Most importantly, the primers for this study were constructed on the basis of complete mitochondrial genome data, generated via 2–6 overlapping long range PCRs, from each one individual of the three species. The comparison of HVI sequences as derived from the HVI primer pair with complete mitochondrial genome sequences revealed no inconsistent positions in these three individuals.

To prevent cross-species contamination, benches and plastic ware were cleaned with 10% bleach and sterile water, and then exposed to UV light for 30 min. The surface of muscle samples was also exposed to UV light (30 min). DNA extraction, PCR amplification and sequencing was conducted in separate laboratories and repeated with random samples after several months. Further, negative controls were clean and sequences from independent analyses were identical.

### Mitochondrial DNA Diversity, Phylogeny and Population Structure

Sequences were aligned using ClustalX [23] and rechecked by eye. Haplotypes were likewise identified with ClustalX. Pairwise sequence differences between haplotypes were calculated using Mega 2.1 [24] and genetic diversity within populations was estimated by haplotype (*h*) and nucleotide diversities (*π*) [25] in DnaSP 4.10 [26]. For phylogenetic reconstructions, we performed maximum-likelihood (ML) and maximum-parsimony (MP) analyses using PAUP* 4.0 [27] and Bayesian analysis in MrBayes 3.0 [28]. The sequences from *T. delacouri* and *T. obscurus* were used as outgroups. MODELTEST 3.06 [29] was run to determine the appropriate model of sequence evolution in a likelihood ratio test framework. In MP analyses, gaps were treated as a fifth state. Bootstrap analyses were performed with 5,000 replicates for MP and 100 full heuristic replicates for ML. For Bayesian phylogenetic inference, four Markov chain Monte Carlo (MCMC) runs were performed for 100,000 generations, sampling every ten generations. The initial 5% of trees were discarded as burn-in. Finally, a minimum spanning network [30] and a median joining network were constructed with TCS 1.13 [31] and Network 4.5.1.6 [32], respectively.

### Divergence Time Estimation

Molecular dating was conducted with BEAST 1.5.3 [33] with a relaxed-clock MCMC approach. As modern Bayesian methods allow for the incorporation of a prior distribution of ages, two calibration points based on Perelman et al. [34] were applied as log-normal or normal priors to constrain the age of the following nodes: (1) the divergence between *T. obscurus* and the *T. francoisi* group was calibrated using a log-normal distribution so that the earliest possible sampled age corresponds to 2.40 (95% highest posterior density [HPD], 1.57–3.23) million years ago (mya), and (2) a normal distribution with mean of 0.64 mya and a standard deviation (SD) of 0.213 for the time to most recent common ancestor (TMRCA) of *T. delacouri* and the ancestor of *T. francoisi*, *T. leucocephalus and T. poliocephalus*. We applied these molecular-based calibration points, because no fossil data are available. The uncorrelated log-normal model was used to estimate substitution rates for all nodes in the tree with uniform priors on the mean (0, 100) and standard deviation (0, 10) of this model. In addition, we employed the Yule process of speciation as the tree prior with the ingroup assumed to be monophyletic with respect to outgroups. Each BEAST analysis consisted of 20 million generations with a random starting tree and sampling every 1,000 generations. Log files from each run were imported into Tracer 1.5 [35] and trees sampled from the first 1 million generations were discarded. Analysis of these parameters in Tracer suggested that the number of MCMC runs was adequate, with effective sample sizes (ESSs) of all parameters often exceeding 200, and Tracer plots showing strong equilibrium after discarding the burn-in. Tree files from the individual runs were combined using LogCombiner 1.5.3. The maximum-clade credibility tree topology and mean node heights were calculated from the posterior distribution of the trees and posterior probabilities ≥0.95 were considered as statistically significant (i.e. “strong”) clade support [36]. Final summary trees were calculated with TreeAnnotator 1.5.3 and viewed in FigTree 1.2.2 [37].

### Historical Demographic Events

Using the full data set, we estimated major demographic changes using BEAST. The Bayesian Skyline Plot (BSP) model was applied to examine a number of different population sizes through time and to run a smoothing procedure to visualize historical population size changes [33]. Standard MCMC sampling is used by BSP to estimate posterior distribution of effective population size through time from a sample of gene sequences, which gives a specified nucleotide-substitution model. Compared with previous methods, the BSP includes credibility intervals for the estimated effective population size at every point in time, back to the most recent common ancestor of the gene sequences [33]. The prior setting was the same as described in the Bayesian analysis above.

To test the hypothesis of demographic expansion, the population parameter *θ* = 2 *N*_ef_ *μ*, where *N*_ef_ is the female effective population size and *μ* is the mutation rate per site per generation, was estimated using π according to the relationship E(θ) = π [38] and using Watterson’s [39] point estimator, *θ*_W_. The estimator π uses the recent population as the inference population, whereas *θ*_W_ uses the historical population as the inference population. We also calculated the maximum likelihood estimates of *θ* for variable population sizes, denoted here as *θ*_var_, jointly with the growth parameter g using the program FLUCTUATE 1.4 [40], which uses genealogical information in the data and applying *θ*_W_ as a starting parameter for MCMC simulations. Stability of the parameter estimation was ensured by conducting ten short MCMC runs of 4,000 steps each and five long chains of length 400,000, with a sampling increment of 20 and one independent re-run. Secondly, a mismatch analysis was conducted using ARLEQUIN 3.0 [41] under a model of population expansion. The overall validity of the estimated demographic model was evaluated by the tests of raggedness index (*Hri*) [42] and the sum of squared differences (SSD) [43]. Significance of *Hri* and SSD was assessed by parametric bootstrapping (10,000 replicates) and a significant value was taken as evidence for departure from the estimated demographic model of sudden population expansion. Third, Tajima’s *D*, Fu and Li’s D* [44], Fu’s *Fs* [45] and Ramos-Onsins and Rozas’s *R_2_* [46] tests for mutation/drift equilibrium were performed in DnaSP and ARLEQUIN with 10,000 simulations. If a population expansion was detected, we estimated its putative age according to the following equation modified from Harpending et al. [47] and recently applied in a study of Japanese macaques (*Macaca fuscata*) [48]: τ =  mlt (equation 1), where τ is the time after expansion in mutational units, m is the mean divergence rate per nucleotide per year, l is the sequence length, and t is the number of years after the expansion episode.

### Population Spatial Structure Analysis

The spatial analysis of molecular variance conducted with SAMOVA 1.0 [49] was used to identify groups of sampling locations, which are geographically and genetically homogeneous and maximally differentiated from each other. This approach relies on a technique of analysis of molecular variance (AMOVA) [41]. However, in contrast to conventional AMOVA, SAMOVA does not require an a priori definition of groups, allowing instead the groups to emerge from the data. The most likely number of groups was identified by running SAMOVA with 2–16 groups and choosing the partition scheme with the highest *φ*_CT_ value.

### Analysis of Isolation by Distance (IBD) and Isolation by Barrier (IBB)

To visualize the spatial distribution of landscapes, we collected SPOT5 satellite imagery of the year 2005 (China Remote Sensing Satellite Ground Station) and developed a vegetation-mapping model with the software ARCGIS (Environmental Systems Research Institute). Karst scrub was identified as suitable habitat for langurs [50]. Because *T. francoisi* and *T. leucocephalus* are separated by the Ming River and Zuo River, and both are wider than 100 m, rivers with a width of more than 100 m were lined out in the map and regarded as putative barriers for langurs. Mantel tests [51] were performed to test the significance of regression between pairwise genetic distances expressed as (PiXY-(PiX+PiY)/2) against the Euclidean geographical distance [52]. To estimate the effect of barriers (habitat gaps, rivers, open sea) to gene flow, a categorical matrix was generated describing the number of habitat gaps and rivers between the sampling lots. Then this matrix was used in further Mantel tests to determine whether it co-varied with genetic distance [53]. Barriers and geographical distance were not independent, because langur groups separated by barriers were usually farther apart from each other. Thus, a partial Mantel test [54] was also performed to assess how much genetic differentiation could be attributed to a barrier after controlling for the effect of Euclidean geographical distance. Mantel and partial Mantel tests were performed in ARLEQUIN with 10,000 iterations to determine the statistical significance.

## Results

### Phylogenetic Analysis and Divergence Time Estimation

**Figure 3:**
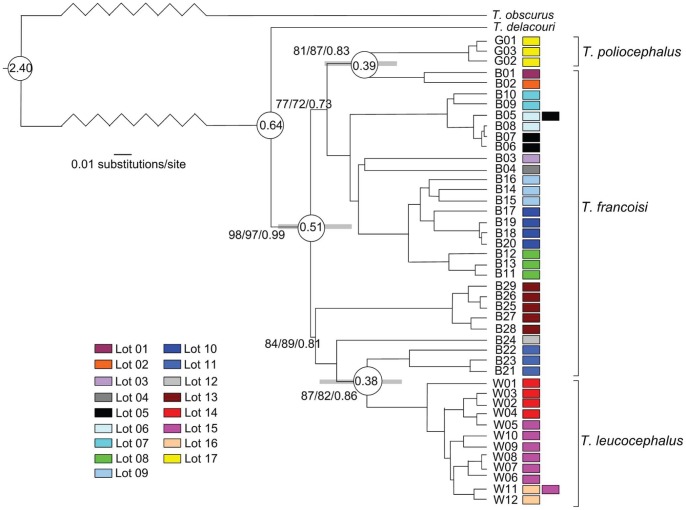
Phylogenetic relationships among *T. francoisi, T. leucocephalus* and *T. poliocephalus* haplotypes. Labels refer to haplotype identification numbers (see Table S1). Values above branches indicate support for each node based on ML/MP/Bayesian algorithms, respectively. Bootstrap values <50% are not shown. Divergence age estimates for major nodes are depicted in circles along with their 95% credibility intervals (grey bars). Sampling lots are presented as colored rectangles.

**Figure 4:**
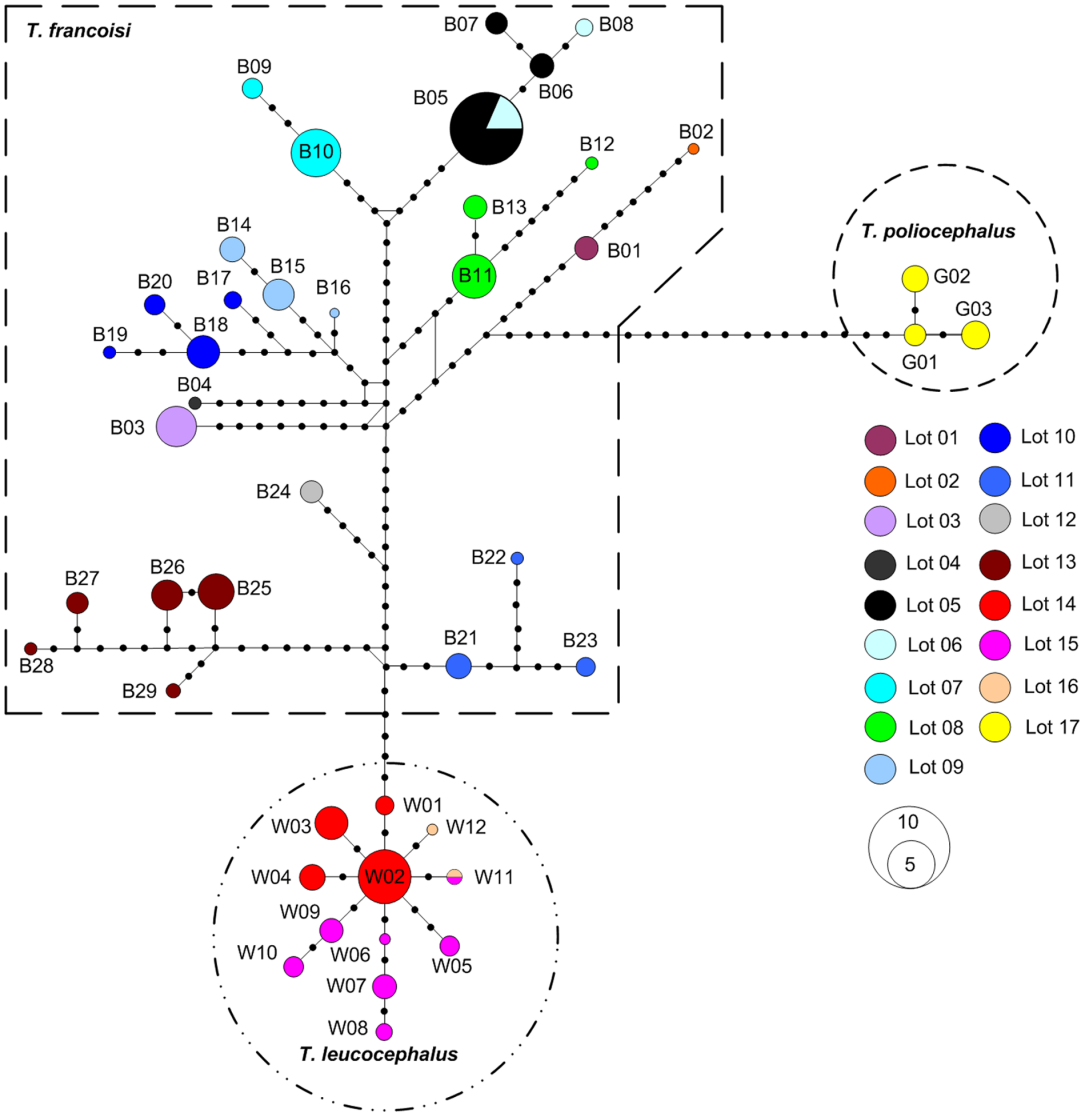
Minimum-spanning network for *T. francoisi, T. leucocephalus* and *T. poliocephalus* haplotypes. Each circle represents a haplotype and the diameter scales to haplotype frequency. Mutational steps are represented by black dots on lines connecting haplotypes. Sampling lots are presented as colored circles.

In the alignment comprising 251 individuals we found 72 variable sites, including 14 transversions (tv) and 61 transitions (ts) (both ts and tv at the 3^rd^, 193^rd^ and 205^th^ site). We defined 29 haplotypes (B01–B29) in *T. francoisi*, 12 haplotypes (W01–W12) in *T. leucocephalus* and three haplotypes (G01–G03) in *T. poliocephalus* (HQ613913–HQ613957, Table S1). The HKY+I+G model was identified as the best-fitting model, with a gamma shape parameter of 1.0035, a ts/tv ratio of 5.71 and base frequencies of A = 0.3226, C = 0.2148 and G = 0.1366. All phylogenetic analyses resulted in almost identical tree topologies (Figure 3). Haplotypes of *T. leucocephalus* (W01–W12) and *T. poliocephalus* (G01–G03) formed monophyletic clades, which were nested within a polyphyletic *T. francoisi* (B01–B29) clade. The network shows a similar pattern (Figure 4). According to Bayesian divergence age estimations, *T. leucocephalus* and *T. poliocephalus* separated from *T. francoisi* populations 0.46-0.27 mya and 0.50-0.25 mya, respectively (Figure 3).

### Population Demographic History

**Figure 5:**
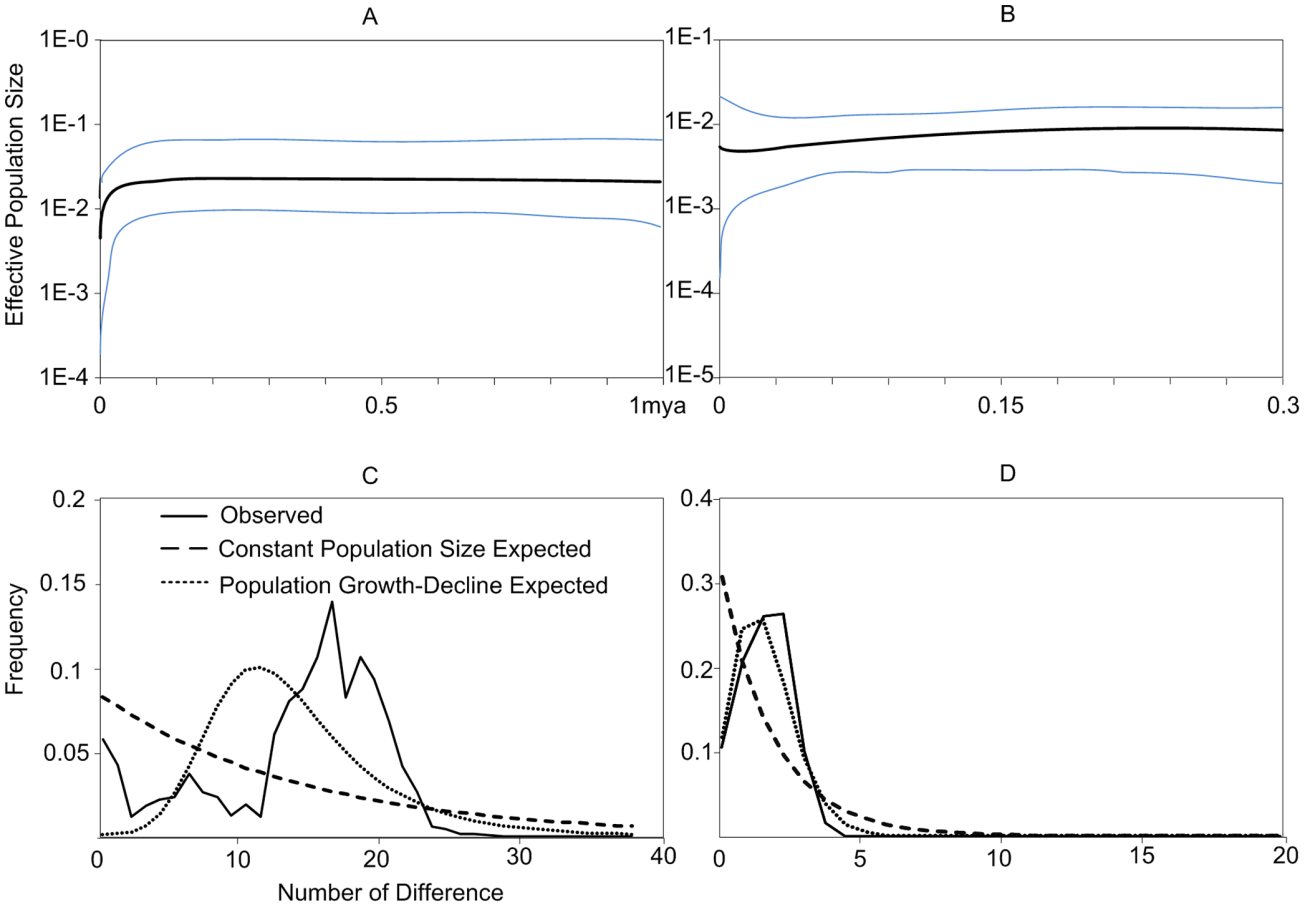
Bayesian skyline plot of past demographic trends and mismatch distributions based on haplotypes of *T. francoisi* (A and C) and *T. leucocephalus* (B and D). In a) and b), x-axis represents time in mya; y-axis represents estimated population size (units = *Neτ*, the product of effective population size and generation time in years, log-transformed). The mean estimate and the 95% HPD limits are indicated as black and blue lines, respectively. In c) and d), the frequencies of pairwise differences haplotypes are shown.

For *T. francoisi*, the BSP suggested a sharp decrease in population size since 0.05 million years (Figure 5a), which is supported by the non-optimistic population growth parameter g (−7.74±12.98) (Table 1). Similarly, Tajima’s *D* and Fu’s *Fs* were estimated at 0.656 (P = 0.802) and 2.872 (P = 0.810), respectively (Table 1), and thus, indicating no population expansion. Consistent with these results, the mismatched distribution revealed an atypical shape of distribution (Figure 5c).

**Table 1:** Summary of haplotype diversity (*h*), nucleotide diversity (*π*), test of selective neutrality (Tajima’s *D*, Fu’s *F_s_*, Fu and Li’s D^*^ and Fu and Li’s F^*^) and population parameters of theta (θ) and growth parameter (g) of HVI region sequences of *T. francoisi*, *T. leucocephalus* and *T. poliocephalus.*

Taxon	*T. francoisi*	*T. leucocephalus*	*T. poliocephalus*
Individuals	178	54	19
Haplotypes	29	12	3
*h*	0.952±0.005	0.878±0.015	0.678±0.051
*π*	0.034±0.001	0.005±0.0003	0.003±0.0002
*D*	0.656(*P* = 0.802)	0.010(*P* = 0.547)	1.811(*P* = 0.982)
*F_s_*	2.872(*P* = 0.810)	−1.001(*P* = 0.371)	1.185(*P* = 0.709)
D^*^	0.491(*P*>0.10)	1.378(*P*>0.05)	1.181(*P*>0.05)
F^*^	1.761(*P*<0.05)	1.072(*P*>0.10)	1.290(*P*>0.10)
θ	0.006±0.003	0.10±0.001	0.001±0.001
g	−7.74±12.98	524.99±197.92	55.23±478.01
SSD	0.027(*P* = 0.013)	0.0071(*P* = 0.084)	0.2951(*P* = 0.004)
*Hri*	0.272(*P* = 0.005)	0.0518(*P* = 0.227)	0.2719(*P* = 0.033)
*R_2_*	0.039(*P* = 0.383)	0.1074(*P* = 0.000)	0.3836(*P* = 0.163)

Values of SSD, *Hri* and *R_2_* are also showed.

For *T. leucocephalus,* a large population growth parameter g (524.99±197.92) and a relatively high *θ*_var_ (0.10±0.001) were found (Table 1). The bell-shaped mismatch distribution (Figure 5d) indicates population expansion in the past, and *τ = *2.33 (95% confidence interval = 1.80–3.07) and the time after expansion was estimated at 0.15-0.06 mya. A recent population expansion for *T. leucocephalus* was also suggested by the BSP (Figure 5b).

### Population Spatial Structure, IBD and IBB Analysis

Haplotypes displayed very strong geographical specificity, consistent with population clustering based on geographic partitioning. Only one (B05) of the 29 *T. francoisi* haplotypes was shared among different sample locations, while all others were specific for their lots (Figure 3, Table S1). For *T. leucocephalus*, haplotypes also displayed strong local homogeneity and population structure. Haplotypes W01–W04 were confined to Lot 14, W05–W11 to Lot 15 and W11–W12 to Lot 16, thus, only W11 was shared among two lots. Considering all the populations of *T. francoisi*, *T. leucocephalus* and *T. poliocephalus*, the SAMOVA procedure based on the pairwise differences yielded a maximized *φ*_CT_ (0.833, *P*<0.0001) for the 12 groups: [Lot01, Lot02] [Lot03] [Lot04] [Lot05, Lot06, Lot07] [Lot08] [Lot09] [Lot10] [Lot11] [Lot12] [Lot13] [Lot14, Lot15, Lot16] [Lot17]. These results suggested that this pattern is the most parsimonious geographical subdivision. 83.34% of the genetic diversity is found between groups, 7.39% existed among sampling lots within groups and only 9.27% existed within sampling lots.

Genetic distances among groups were calculated by pairwise (PiXY-(PiX+PiY)/2) and the Euclidean geographical distances were estimated by GIS analysis (Table S2). Pairwise (PiXY-(PiX+PiY)/2) ranged from 0.536–31.700 and Euclidean geographical distance spanned from 42.28–2492.28 km. A categorical matrix was generated to describe whether the sampling lots are connected by habitat or separated by barriers. Sampling lots in connected habitat fragments had a categorical distance of 0. For sampling lots that were isolated by one or more barriers, the categorical distances between them was equal to the number of barriers. Isolation-by-distance analyses using the Mantel test revealed that 24.5% of the genetic distance among sampling groups can be explained by Euclidean geographical distance when the complete study area is considered (*r* = 0.49, *P*<0.01). The Mantel test for the effect of barriers on genetic distance confirmed a strong influence on gene flow and explained 59.3% of the genetic differentiation among sampling locations (*r* = 0.52, *P<*0.01). The partial Mantel test revealed a significant positive correlation between genetic distance and the presence of barriers (*r* = 0.23, *P* = 0.01) after controlling for the effect of Euclidean geographical distance. 58.4% of genetic distance was determined by the presence of barriers. Thus, we conclude that barriers as habitat gaps, rivers or open sea form a stronger barrier to gene flow than Euclidean geographical distance alone.

## Discussion

### Evolutionary History and Population Demography of the three Langurs

The distribution range of *T. francoisi* is much larger than that of the other two species and we also found more haplotypes. *T. leucocephalus* is confined to a narrow triangular karst hill region of 200 km^2^ in southern Guangxi Province (107–108°E, 22°06′–22°42′N), China and is separated from *T. francoisi* by the Ming and Zuo Rivers (Figure 2). Haplotypes found in white-headed langurs form a monophyletic clade that diverged from *T. francoisi* 0.46-0.27 mya (Figure 3) and population demographic analyses of *T. leucocephalus* indicate historical expansion 0.15-0.06 mya. Hence, relatively low genetic variation and recent population expansion suggests that *T. leucocephalus* emerged from a small founder population.

During phases of the Middle Pleistocene, between 0.5-0.4 mya, the current course of the Ming River was formed and the Zuo River became wider [55], [56]. Accordingly, ancestral *T. leucocephalus* populations became physically separated from *T. francoisi* and a new phenotype emerged most likely via new mutations in a small population. Subsequently, *T. leucocephalus* experienced a population expansion, which might have contributed to the accumulation of the new pelage coloration.

Noteworthy, *T. francoisi* and *T. leucocephalus* are able to hybridize. Hu et al. [11] and Que et al. [57] reported a female hybrid in Nanning Zoo, but it died because of a missing kidney, which could be the result of outbreeding depression [58]. Furthermore, in 2006 a confiscated *T. francoisi* female was accidentally released into the range of *T. leucocephalus* (Lot 14) and she hybridized with a *T. leucocephalus* male producing several offspring (Deng, personal communication). All hybrid offspring showed pelage coloration characteristics of *T. leucocephalus*, which might indicate that white pelage on the head, crest and tail tip is a dominant character.

As a result of this release, the *T. francoisi* haplotype B19 was introduced in Lot 14, which we were able to confirm in our study. However, to avoid confusion we excluded this data from further analysis. If other cases of human-mediated gene flow among populations occurred in the past remains speculative. However, haplotypes in all three species show strong geographical specificity and only two haplotypes were found in more than one lot (*T. francoisi* haplotype B05 in Lots 5 and 6, *T. leucocephalus* haplotype W11 in Lots 15 and 16), suggesting that if human-mediated gene flow occurred at all (besides the case mentioned above), it was very limited.

Also for the golden-headed langur, a relatively recent divergence from *T. francoisi* was estimated (0.50-0.25 mya, Figure 3). The separation of *T. poliocephalus* on Cat Ba Island in Halong Bay from all other taxa of the species group seems to be most likely caused by a larger gap of suitable karst habitat on the mainland and open sea. Particularly the lack of suitable habitat between *T. francoisi* and *T. poliocephalus* of more than 200 km might be the main reason for the interruption of gene flow between them. Open sea as barrier to gene flow might also have been effective; although the sea between Cat Ba Island and the Vietnamese mainland is less than 20 m deep [59] and repeated connections between both landmasses emerged in the last 0.5 million years [60]. However, the repeated submersion and exposure of soils on the shelf may have affected soil structure and fertility significantly, and consequently the structure of forest communities [61]. As shown for the Sunda shelf, migration between islands was extremely limited, although they were repeatedly connected during the Quaternary, the last time during the last glacial period [62]–[64].

In summary, both *T. leucocephalus* and *T. poliocephalus* seem to have a similar evolutionary history. Both might be recent, but independent descendents from *T. francoisi* and both are the result of most likely small founder populations; findings that support our hypothesis 1. After the split from *T. francoisi*, both have evolved different pelage colorations within a relatively short time period, in particular on the head and on the shoulders. Since *T. francoisi* populations show nearly no variability in pelage coloration, pelage coloration in *T. leucocephalus* and *T. poliocephalus* is most likely the result of new independent genetic mutations after the split from *T. francoisi* and not of the fixation of different characters derived from an ancestral polymorphism.

### Population Structure and the Effects of Isolation

The distribution of haplotypes displays local homogeneity, implying strong population structure and genetic differentiation for all three species. In *T. francoisi*, all sampling lots are separated from each other by habitat gaps, rivers or geographical distance. Haplotypes also display very strong geographical specificity, consistent with clustering of patches based on geographical partitioning. Fine-scale population structure is common in large mammals and influenced by various factors [66]–[67]. Our study shows significant mitochondrial differentiation among all three species and within *T. francoisi* and *T. leucocephalus* populations. This suggests that habitat gaps, rivers and open sea are major physical barriers for gene flow in structuring genetic variation at inter- and intra-specific levels. SAMOVA, median-joining networks, IBD and IBB analyses clearly indicated that several major haplotype groups of *T. francoisi* and *T. leucocephalus* are restricted to habitats, fragmented by river catchments. Accordingly, incomplete lineage sorting seems to be an unlikely explanation for the observed pattern, because lineage sorting should be random with respect to geography [65]. However, it remains unclear whether the geographic structuring of mitochondrial haplotypes observed in this study is generally true for the genome because any single locus can give a non-representative result.

Besides ecological factors, also social structure largely influences population genetic structure, which is mediated mainly by social behavior (reproductive skew, dispersal, fission and fusion patterns) [53], [68]–[71]. In most colobines, including species of the genus *Trachypithecus*, females tend to stay in their natal groups (female philopatry) and males migrate at time of sexual maturity [15], [16], leading to significant population differentiation when solely maternally-inherited markers as the herein applied HVI region are studied [68], [71]. Thus, male-mediated gene flow is not captured in our study and accordingly to fully understand the evolutionary history of these three species further investigations should apply nuclear loci as well.

### Conservation Implications

*T. francoisi* is classified as “Endangered” and *T. poliocephalus* and *T. leucocephalus* as “Critically Endangered” by the IUCN Red List [12]–[14]. Accordingly, conservation measures are urgently required to save these species from extinction. In last centuries, *T. francoisi* was widely distributed in karst forests of tropical and subtropical south-western China (Chongqin, Guizhou and Guangxi provinces) and northern Vietnam [12]. However, *T. francoisi* has experienced a dramatic decline of 85% in population size and 70% in distribution. Prior to 1980, the species was found in 23 different counties in China and numbered 8,000–10,000 animals. In 2007, it was estimated that there were only 1,900–2,150 animals left in the wild [72]. The situation for *T. leucocephalus* and *T. poliocephalus* is even more dire with estimates of only 580–620 and a maximum of 70 animals in the wild, respectively [12]–[14], [50], [73]. Recent attention on the conservation of *T. leucocephalus* in Lot 14 and Lot 15 has resulted in population increase [74], [75], but in other forest patches it continued to decline or even became extinct. For the golden-headed langur, the population dropped from 2,500–2,800 individuals in the 1960s to 53 individuals in 2000 [14].

Our study shows that these langurs face a serious problem: habitat fragmentation and limited if any gene flow. We posit that geographical structuring of the populations is a direct issue for conservation. Species characterized by limited mobility and strong population genetic structure, such as the three herein studies species, are more prone to suffer from substantial loss of genetic diversity as a result of local extinction [76]–[78]. Additionally, haplotypes in the different forest patches form isolated groups and 83.3% of the genetic diversity within *T. francoisi* (including *T. leucocephalus* and *T. poliocephalus*) is found between these lots. Subpopulations that are highly divergent must be protected so as to safe guard their unique genetic diversity.

### Conclusions

For all these three langurs, habitat is fragmented by habitat gaps, river catchments and sea barriers. Our study indicates that such barriers have played a key role in shaping the present-day population structure of species and populations, and that gene flow between them appears to be strongly impeded by these barriers. Mutations causing pelage coloration changes might have occurred in ancestral *T. leucocephalus* and *T. poliocephalus* populations, which became later fixed due to isolation. Thus, physical isolation could have provided the evolutionary potential for the divergence of different species, and even resulted in speciation and the significant diversity seen in Asian colobines today. However, since we analyzed only mitochondrial DNA, male-mediated gene flow is not captured by our study. Thus, to fully understand the evolutionary history of these and other species, nuclear loci should be included in future studies as well.

## Supporting Information

Table S1 **Summary of haplotype distributions of HVI region sequences of** ***T. francoisi*** **,** ***T. leucocephalus*** **and** ***T. poliocephalus.***
(DOC)

Table S2 **Genetic and geographical distances between the 17 Lots (a) and categorical distance matrix describing presence or absence of habitat gaps among sampling groups (b).**
(DOC)
