# Supplementary material for: Relatively Recent Evolution of Pelage Coloration in Colobinae: Phylogeny and Phylogeography of Three Closely Related Langur Species
Source: PLoS One. 2013 Apr 17;8(4):e61659. doi: 10.1371/journal.pone.0061659 (PMC3629164; doi:10.1371/journal.pone.0061659)
Supplement: Table S1 — Summary of haplotype distributions of HVI region sequences of T. francoisi , T. leucocephalus and T. poliocephalus. (DOC) [file pone.0061659.s001.doc]

**Table S2** (a) Genetic and geographical distances between the 17 Lots. Genetic distance is represented by pairwise (PiXY-(PiX+PiY)/2) (upper diagonal) and Euclidean geographical distance in km (lower diagonal). (b) Categorical distance matrix describing presence or absence of habitat gaps among sampling groups. For sampling groups in connective rivers, the categorical distance between them was 0; when isolated by rivers the categorical distance between them was equal to the number of gaps and barriers.

| (a) Genetic and geographical distances | | | | | | | | | | | | | | | | | | | | | | | | | | | | | | | | |
| --- | --- | --- | --- | --- | --- | --- | --- | --- | --- | --- | --- | --- | --- | --- | --- | --- | --- | --- | --- | --- | --- | --- | --- | --- | --- | --- | --- | --- | --- | --- | --- | --- |
| Lots | Lot01 | Lot02 | Lot03 | | Lot04 | | Lot05 | | Lot06 | | Lot07 | | Lot08 | | Lot09 | | Lot10 | | Lot11 | | Lot12 | | Lot13 | | Lot14 | | Lot15 | | Lot16 | | Lot17 | |
| Lot01 |  | 1.076 | 0.536 | | 13.031 | | 14.004 | | 14.555 | | 17.157 | | 17.801 | | 17.489 | | 17.449 | | 23.449 | | 14.272 | | 18.625 | | 18.801 | | 19.952 | | 19.135 | | 25.822 | |
| Lot02 | 42.28 |  | 1.695 | | 11.387 | | 12.359 | | 13.026 | | 15.192 | | 15.667 | | 15.359 | | 15.667 | | 21.667 | | 11.667 | | 16.000 | | 16.667 | | 17.817 | | 17.000 | | 25.883 | |
| Lot03 | 357.72 | 288.48 |  | | 12.701 | | 13.674 | | 14.492 | | 17.804 | | 18.029 | | 17.961 | | 17.648 | | 23.648 | | 15.648 | | 18.648 | | 19.029 | | 20.179 | | 19.362 | | 27.578 | |
| Lot04 | 461.52 | 392.28 | 173.15 | |  | | 6.937 | | 5.897 | | 16.641 | | 13.053 | | 15.301 | | 11.053 | | 17.053 | | 12.053 | | 12.053 | | 18.053 | | 19.169 | | 18.067 | | 30.603 | |
| Lot05 | 2103.94 | 2065.38 | 2053.86 | | 1915.38 | |  | | 2.593 | | 15.391 | | 13.915 | | 15.830 | | 14.026 | | 16.137 | | 12.804 | | 10.915 | | 16.804 | | 17.954 | | 17.088 | | 27.354 | |
| Lot06 | 2019.24 | 1996.14 | 1926.9 | | 1788.48 | | 323.16 | |  | | 15.170 | | 13.753 | | 13.446 | | 14.390 | | 18.390 | | 10.753 | | 9.844 | | 16.753 | | 17.903 | | 16.516 | | 26.590 | |
| Lot07 | 1742.28 | 1743.48 | 1719.24 | | 1592.28 | | 369.24 | | 461.52 | |  | | 10.150 | | 9.472 | | 15.775 | | 15.150 | | 15.088 | | 18.588 | | 8.775 | | 9.925 | | 10.308 | | 24.298 | |
| Lot08 | 1188.48 | 1121.52 | 906.29 | | 761.52 | | 1523.14 | | 1292.28 | | 1280.76 | |  | | 12.211 | | 16.000 | | 18.000 | | 18.000 | | 19.000 | | 13.000 | | 14.150 | | 14.533 | | 28.287 | |
| Lot09 | 576.90 | 523.86 | 403.86 | | 230.76 | | 1673.1 | | 1534.62 | | 1361.52 | | 600.91 | |  | | 16.359 | | 16.359 | | 15.692 | | 17.248 | | 12.544 | | 13.694 | | 12.451 | | 23.831 | |
| Lot10 | 923.12 | 841.14 | 646.14 | | 484.62 | | 1580.76 | | 1396.14 | | 1303.86 | | 265.38 | | 346.14 | |  | | 6.000 | | 18.000 | | 19.000 | | 19.000 | | 20.150 | | 19.573 | | 27.550 | |
| Lot11 | 750.7 | 654.24 | 380.76 | | 311.52 | | 2076.98 | | 1915.38 | | 1788.48 | | 715.38 | | 484.62 | | 519.24 | |  | | 19.000 | | 21.000 | | 19.000 | | 20.150 | | 19.573 | | 23.550 | |
| Lot12 | 542.28 | 483.48 | 196.14 | | 126.93 | | 1996.14 | | 1857.72 | | 1684.62 | | 726.93 | | 323.14 | | 496.14 | | 196.14 | |  | | 14.000 | | 19.000 | | 20.150 | | 19.013 | | 25.550 | |
| Lot13 | 900.95 | 830.76 | 542.28 | | 496.14 | | 2134.62 | | 1926.94 | | 1857.72 | | 657.72 | | 611.52 | | 542.28 | | 173.1 | | 369.24 | |  | | 20.000 | | 21.150 | | 18.893 | | 28.550 | |
| Lot14 | 1050.67 | 980.76 | 692.28 | | 576.97 | | 1926.92 | | 1707.72 | | 1684.62 | | 415.38 | | 603.8 | | 380.76 | | 380.76 | | 519.24 | | 265.38 | |  | | 1.150 | | 1.653 | | 30.550 | |
| Lot15 | 1163.16 | 1084.62 | 807.72 | | 692.28 | | 1973.17 | | 1742.28 | | 1742.28 | | 450.55 | | 715.38 | | 496.14 | | 461.52 | | 623.16 | | 323.11 | | 103.86 | |  | | 0.804 | | 31.700 | |
| Lot16 | 866.52 | 773.12 | 507.72 | | 403.86 | | 1984.62 | | 1809.7 | | 1696.14 | | 542.28 | | 496.14 | | 392.28 | | 173.15 | | 288.48 | | 150.34 | | 207.72 | | 87.33 | |  | | 30.563 | |
| Lot17 | 891.99 | 830.76 | 600.35 | 634.62 | | 2492.28 | | 2319.24 | | 2192.28 | | 1084.62 | | 865.38 | | 923.15 | | 403.86 | | 519.24 | | 415.38 | | 692.28 | | 726.94 | | 530.76 | |  | |  |
| (b) Categorical distance matrix | | | | | | | | | | | | | | | | | | | | | | | | | | | | | | | | |
| Lots | Lot01 | Lot02 | Lot03 | | Lot04 | | Lot05 | | Lot06 | | Lot07 | | Lot08 | | Lot09 | | Lot10 | | Lot11 | | Lot12 | | Lot13 | | Lot14 | | Lot15 | | Lot16 | | Lot17 | |
| Lot01 | 0 |  |  | |  | |  | |  | |  | |  | |  | |  | |  | |  | |  | |  | |  | |  | |  | |
| Lot02 | 1 | 0 |  | |  | |  | |  | |  | |  | |  | |  | |  | |  | |  | |  | |  | |  | |  | |
| Lot03 | 2 | 1 | 0 | |  | |  | |  | |  | |  | |  | |  | |  | |  | |  | |  | |  | |  | |  | |
| Lot04 | 5 | 4 | 2 | | 0 | |  | |  | |  | |  | |  | |  | |  | |  | |  | |  | |  | |  | |  | |
| Lot05 | 21 | 20 | 21 | | 18 | | 0 | |  | |  | |  | |  | |  | |  | |  | |  | |  | |  | |  | |  | |
| Lot06 | 22 | 22 | 18 | | 16 | | 5 | | 0 | |  | |  | |  | |  | |  | |  | |  | |  | |  | |  | |  | |
| Lot07 | 24 | 23 | 20 | | 19 | | 7 | | 5 | | 0 | |  | |  | |  | |  | |  | |  | |  | |  | |  | |  | |
| Lot08 | 14 | 12 | 6 | | 4 | | 11 | | 7 | | 13 | | 0 | |  | |  | |  | |  | |  | |  | |  | |  | |  | |
| Lot09 | 5 | 3 | 4 | | 4 | | 13 | | 10 | | 14 | | 5 | | 0 | |  | |  | |  | |  | |  | |  | |  | |  | |
| Lot10 | 9 | 7 | 6 | | 2 | | 15 | | 11 | | 13 | | 4 | | 4 | | 0 | |  | |  | |  | |  | |  | |  | |  | |
| Lot11 | 6 | 3 | 1 | | 2 | | 21 | | 15 | | 23 | | 6 | | 7 | | 5 | | 0 | |  | |  | |  | |  | |  | |  | |
| Lot12 | 7 | 6 | 3 | | 3 | | 21 | | 15 | | 22 | | 7 | | 7 | | 5 | | 2 | | 0 | |  | |  | |  | |  | |  | |
| Lot13 | 9 | 7 | 4 | | 4 | | 22 | | 16 | | 19 | | 8 | | 8 | | 5 | | 4 | | 2 | | 0 | |  | |  | |  | |  | |
| Lot14 | 12 | 10 | 4 | | 3 | | 20 | | 14 | | 16 | | 5 | | 10 | | 1 | | 4 | | 3 | | 3 | | 0 | |  | |  | |  | |
| Lot15 | 11 | 9 | 3 | | 3 | | 21 | | 15 | | 17 | | 6 | | 9 | | 2 | | 3 | | 2 | | 2 | | 1 | | 0 | |  | |  | |
| Lot16 | 11 | 9 | 3 | | 3 | | 21 | | 15 | | 17 | | 6 | | 9 | | 2 | | 3 | | 2 | | 2 | | 1 | | 0 | | 0 | |  | |
| Lot17 | 5 | 3 | 3 | | 7 | | 23 | | 20 | | 23 | | 13 | | 10 | | 7 | | 4 | | 3 | | 3 | | 7 | | 6 | | 6 | | 0 | |
