# Supplementary material for: Relatively Recent Evolution of Pelage Coloration in Colobinae: Phylogeny and Phylogeography of Three Closely Related Langur Species
Source: PLoS One. 2013 Apr 17;8(4):e61659. doi: 10.1371/journal.pone.0061659 (PMC3629164; doi:10.1371/journal.pone.0061659)
Supplement: Table S2 — Genetic and geographical distances between the 17 Lots (a) and categorical distance matrix describing presence or absence of habitat gaps among sampling groups (b). (DOC) [file pone.0061659.s002.doc]

**Table S1** Summary of haplotype distributions of HVI region sequences of *T. francoisi*, *T. leucocephalus* and *T. poliocephalus* (HQ613913-HQ613957).

| Taxon  Lot  Haplotype | *T. francoisi* | | | | | | | | | | | | | *T. leucocephalus* | | | *T. poliocephalus* |
| --- | --- | --- | --- | --- | --- | --- | --- | --- | --- | --- | --- | --- | --- | --- | --- | --- | --- |
|  | 01 | 02 | 03 | 04 | 05 | 06 | 07 | 08 | 09 | 10 | 11 | 12 | 13 | 14 | 15 | 16 | 17 |
| B01 | 2 |  |  |  |  |  |  |  |  |  |  |  |  |  |  |  |  |
| B02 |  | 11 |  |  |  |  |  |  |  |  |  |  |  |  |  |  |  |
| B03 |  |  | 1 |  |  |  |  |  |  |  |  |  |  |  |  |  |  |
| B04 |  |  |  | 2 |  |  |  |  |  |  |  |  |  |  |  |  |  |
| B05 |  |  |  |  | 10 | 2 |  |  |  |  |  |  |  |  |  |  |  |
| B06 |  |  |  |  | 4 |  |  |  |  |  |  |  |  |  |  |  |  |
| B07 |  |  |  |  | 3 |  |  |  |  |  |  |  |  |  |  |  |  |
| B08 |  |  |  |  |  | 4 |  |  |  |  |  |  |  |  |  |  |  |
| B09 |  |  |  |  |  |  | 4 |  |  |  |  |  |  |  |  |  |  |
| B10 |  |  |  |  |  |  | 17 |  |  |  |  |  |  |  |  |  |  |
| B11 |  |  |  |  |  |  |  | 18 |  |  |  |  |  |  |  |  |  |
| B12 |  |  |  |  |  |  |  | 2 |  |  |  |  |  |  |  |  |  |
| B13 |  |  |  |  |  |  |  | 5 |  |  |  |  |  |  |  |  |  |
| B14 |  |  |  |  |  |  |  |  | 7 |  |  |  |  |  |  |  |  |
| B15 |  |  |  |  |  |  |  |  | 10 |  |  |  |  |  |  |  |  |
| B16 |  |  |  |  |  |  |  |  | 1 |  |  |  |  |  |  |  |  |
| B17 |  |  |  |  |  |  |  |  |  | 2 |  |  |  |  |  |  |  |
| B18 |  |  |  |  |  |  |  |  |  | 5 |  |  |  |  |  |  |  |
| B19 |  |  |  |  |  |  |  |  |  | 10 |  |  |  |  |  |  |  |
| B20 |  |  |  |  |  |  |  |  |  | 5 |  |  |  |  |  |  |  |
| B21 |  |  |  |  |  |  |  |  |  |  | 8 |  |  |  |  |  |  |
| B22 |  |  |  |  |  |  |  |  |  |  | 3 |  |  |  |  |  |  |
| B23 |  |  |  |  |  |  |  |  |  |  | 5 |  |  |  |  |  |  |
| B24 |  |  |  |  |  |  |  |  |  |  |  | 10 |  |  |  |  |  |
| B25 |  |  |  |  |  |  |  |  |  |  |  |  | 10 |  |  |  |  |
| B26 |  |  |  |  |  |  |  |  |  |  |  |  | 8 |  |  |  |  |
| B27 |  |  |  |  |  |  |  |  |  |  |  |  | 4 |  |  |  |  |
| B28 |  |  |  |  |  |  |  |  |  |  |  |  | 2 |  |  |  |  |
| B29 |  |  |  |  |  |  |  |  |  |  |  |  | 3 |  |  |  |  |
| W01 |  |  |  |  |  |  |  |  |  |  |  |  |  | 3 |  |  |  |
| W02 |  |  |  |  |  |  |  |  |  |  |  |  |  | 10 |  |  |  |
| W03 |  |  |  |  |  |  |  |  |  |  |  |  |  | 8 |  |  |  |
| W04 |  |  |  |  |  |  |  |  |  |  |  |  |  | 5 |  |  |  |
| W05 |  |  |  |  |  |  |  |  |  |  |  |  |  |  | 4 |  |  |
| W06 |  |  |  |  |  |  |  |  |  |  |  |  |  |  | 9 |  |  |
| W07 |  |  |  |  |  |  |  |  |  |  |  |  |  |  | 2 |  |  |
| W08 |  |  |  |  |  |  |  |  |  |  |  |  |  |  | 3 |  |  |
| W09 |  |  |  |  |  |  |  |  |  |  |  |  |  |  | 4 |  |  |
| W10 |  |  |  |  |  |  |  |  |  |  |  |  |  |  | 3 |  |  |
| W11 |  |  |  |  |  |  |  |  |  |  |  |  |  |  | 1 | 1 |  |
| W12 |  |  |  |  |  |  |  |  |  |  |  |  |  |  |  | 1 |  |
| G01 |  |  |  |  |  |  |  |  |  |  |  |  |  |  |  |  | 4 |
| G02 |  |  |  |  |  |  |  |  |  |  |  |  |  |  |  |  | 7 |
| G03 |  |  |  |  |  |  |  |  |  |  |  |  |  |  |  |  | 8 |
